# Assessment of Stunting and Its Effect on Wasting in Children Under Two in Rural Madagascar

**DOI:** 10.3390/children12060686

**Published:** 2025-05-26

**Authors:** Rosita Rotella, María Morales-Suarez-Varela, Agustín Llopis-Gonzalez, José M. Soriano

**Affiliations:** 1 Research Group in Social and Nutritional Epidemiology, Pharmacoepidemiology and Public Health, Department of Preventive Medicine and Public Health, Food Sciences, Toxicology and Forensic Medicine, Faculty of Pharmacy, Universitat de València, Av. Vicent Andrés Estelles s/n, 46100 Burjassot, Spain; rotella@alumni.uv.es (R.R.); agustin.llopis@uv.es (A.L.-G.); 2 Centro Medico-Chirurgico Saint Paul (Change ONG), Andasibe-Ampefy 118, District of Soavinandriana, Itasy Province, Madagascar; 3 Biomedical Research Center in Epidemiology and Public Health Network (CIBERESP), Carlos III Health Institute, Av. Monforte de Lemos 3-5 Pabellón 11 Planta 0, 28029 Madrid, Spain; 4 Observatory of Nutrition and Food Safety for Developing Countries, Food & Health Lab, Institute of Materials Science, University of Valencia, Carrer Catedrático Agustín Escardino 9, 46980 Paterna, Spain; jose.soriano@uv.es; 5 Joint Research Unit on Endocrinology, Nutrition and Clinical Dietetics, University of Valencia-Health Research Institute La Fe, Avda. Fernando Abril Martorell, 106, 46026 Valencia, Spain

**Keywords:** stunting, wasting, infant malnutrition, Madagascar, nutritional programs

## Abstract

Background/Objectives: This study aims to determine the prevalence of stunting in children under two years old and its association with the maternal profile (including anthropometric measurements), care, feeding practices, and socioeconomic level. It also attempts to assess if stunting may contribute to an underestimation of wasting by performing a preliminary speculative analysis using the expected height for age instead of the real observed height of the children. Methods: The study employed a cross-sectional design, examining mother–child pairs in the rural municipality of Ampefy in the Itasy Region of Madagascar, between 2022 and 2023. A total of 437 mother–child (0–24 months) pairs participated in the study. A questionnaire was administered to collect data on the maternal lifestyle. Maternal medical histories were reviewed, and anthropometric parameters of both the mothers and their child were taken by specialized and trained health professionals with multiple years of experience. Results: The prevalence of stunting in children was 57.4% (95% CI: 52.64–62.10). Stunting was associated with maternal anthropometric measurements (*p* < 0.001), maternal education (*p* = 0.004), and breastfeeding (*p* = 0.047), which appears to have a protective effect. The weight-for-length z-score indicated that only 12.4% of the total children were affected by wasting. In the preliminary speculative analysis using the WHO height-for-age standard, the theoretical prevalence of wasting was estimated to be 42.3%, with a considerable prevalence of severe wasting. The main limitations of this study were the possible selection bias, the limitations inherent to the taking of anthropometric measurements in small children, and therefore, the possible misclassification of the children. The use of a theoretical weight-for-length z-score to estimate a theoretical prevalence of wasting using an untested speculative analysis is also a limitation to the validity of the estimation. Conclusions: Stunting affected over half of the children included in the study (57.4%), but the prevalence of wasting was below what was expected, at 12.4%. In the preliminary speculative analysis using the expected height for age, it was estimated that wasting could possibly affect up to 42.3% of the children. This discrepancy, while it cannot be taken as factual due to the nature of the analysis, could serve as a warning that perhaps the elevated rates of stunting may be masking wasting in some children and other forms of nutritional assessments may be needed in areas where stunting is prevalent.

## 1. Introduction

In 1972, J.C. Waterlow [1,2] first proposed the definitions of protein–calorie malnutrition in order to address the need to carry out studies on the prevalence of malnutrition using standardized criteria. The classification was based on three key aspects: quality, severity, and duration. This led to the definition of two ratios as indicators of the past and present nutritional status: the height-for-age (HFA) ratio was defined as “a picture of the past nutritional history”, while the weight-for-height (WFH) ratio was defined as “an index of current nutritional status” [2].

From these definitions, the term “stunting” can be identified by means of an assessment of the child’s length/height in comparison with international standards for their age. The World Health Organization (WHO) defines a child as moderately stunted if their height or length is at or below the −2 standard deviations (SDs) but equal or greater than −3 SDs from the median for the same age and sex. A child may be defined as severely stunted if they have a length/height below the −3 SDs from the median for the same age and sex [3]. The first 1000 days of life are an essential developmental period as stunting, known also as “chronic malnutrition”, often begins in the womb and continues for at least the first two years of post-natal life [4].

The term “wasting” refers to the assessment of a child’s weight in comparison with the international standards for their length/height [3] established by the WHO. The threshold for moderate wasting is defined as a weight at or below the −2 SDs but equal or greater than −3 SDs from the median for the same length/height and sex. A child may be defined as severely wasted if their weight is below the −3 SDs from the median for the same length/height and sex. It is important to note that wasting, also sometimes known as “acute malnutrition”, if left untreated, can last several months, which does not correspond to the usual meaning of “acute” [5,6,7].

In the absence of an adequate intake of energy, the body undergoes a series of physiological adaptations to ensure the necessary provision of fuel to essential organs. The body relies primarily on nutritional reserves, namely adipose tissue and skeletal muscle [8] The progressive reduction of fat and muscle mass, if severe and protracted over time, has the potential to compromise the fuel supply to vital organs. This exposes the malnourished individual to an increased risk of infection and death [9]. Children with severe wasting are often stunted, which suggests that wasting and stunting may have a common cause or that one form of malnutrition may contribute to the development of the other [10,11,12].

The 2023 update of the “Levels and Trends in Child Malnutrition” report [13] indicates that stunting affects 148.1 million or 22.3% of children under the age of five worldwide while 45 million or 6.8% are affected by wasting. Madagascar is among the countries where stunting and wasting rates are worryingly high, particularly in rural areas [14,15].

The objective of this study is to ascertain the prevalence of stunting among children aged 0 to 24 months using their anthropometric measurements and to examine its possible associations with the maternal profile, care, feeding practices, and socioeconomic conditions. Additionally, it aims to evaluate whether stunting can lead to an underestimation of wasting at the community level by performing a preliminary speculative analysis using the expected HFA taken from the WHO’s anthropometrical standards instead of the real observed height of the children.

## 2. Methods

The study was approved by the Ethics Committee of the Universitat de Valencia (Spain) (protocol code Register code: 2089516, with 7 July 2022 as the date of approval) and the Ethics Committee of the St. Paul Medical-Surgical Centre and from Institut d’Enseignement Superieur de Soavinandriana, Universitè d’Antananarivo (Madagascar) (protocol code Register code: 20220197, with 27 October 2022 as the date of approval), adhering to the guidelines in the World Medical Association (2000) Declaration of Helsinki: Ethical Principles for Medical Research Involving Human Subjects, with notes of clarification of 2002 and 2004, the Guidelines on the Practice of Ethics Committees Involved in Medical Research Involving Human Subjects and the Guidelines for the Ethical Conduct of Medical Research Involving Children, revised in 2000 by the Royal College of Paediatrics and Child Health: Ethics Advisory Committee.

Written informed consent was obtained from all participants. Local health center workers verbally translated the informed consent documents, and each participant received an identification number to anonymize the data collected and ensure confidentiality.

### 2.1. Study Design

This was a cross-sectional mother–child study conducted from 1 November 2022 to 31 March 2023 in Ampefy, Itasy Region, Madagascar.

### 2.2. Study Setting

Ampefy is a rural commune with a population of 25,078 divided into 13 smaller administrative units known as fokontany. Fokontany represent the smallest administrative divisions in Madagascar, comprising clusters of small settlements. The population in this region is diverse, with various Malagasy cultural and ethnic groups, although the Merina ethnic group predominates [16,17].

The region has a subtropical climate heavily influenced by monsoon patterns. Madagascar is among the nations most impacted by the climate crisis, and a 2022 UNICEF report reveals that recent severe weather events have exacerbated the country’s already prevalent food insecurity and malnutrition problems [18,19].

### 2.3. Study Population

Potential study participants were identified by Change Onlus NGO staff through the patient registry of the St Paul Medical-Surgical Centre, which serves as the headquarters for the NGO. This approach facilitated the initial selection of women who met the inclusion criteria: (i) women of reproductive ages (WRAs) (12–49 years), (ii) with at least one living child aged 0–24 months, and (iii) breastfeeding at least one child aged 0–24 months. Exclusion criteria included (i) a lack of informed consent, (ii) unreliable responses, and (iii) incomplete questionnaire responses.

After identifying all eligible women from the health center registers, a systematic random sampling method was used to choose the women to invite to participate. Approximately 5000 women formed the pool of potential participants, and 500 were selected to be invited to participate. Of the 500, 437 were able to be invited and all invited women voluntarily agreed to participate and provided written informed consent. This led to 437 mother–child pairs being included in the study.

### 2.4. Data Collection

Data collection commenced in November 2022 and concluded by the end of March 2023. Prior to the start of data collection, a seven-day training course on data collection techniques was conducted to familiarize the research team with the study procedures and tools.

A trained health worker administered a semi-structured, non-pre-validated questionnaire specifically designed for this study aimed to collect data on maternal education levels, attitudes toward antenatal care, dietary habits during pregnancy and breastfeeding, micronutrient intake adequacy, dietary diversity [20], and sociodemographic variables via a tablet during a 20-min face-to-face interview.

Initially formulated in English, the questionnaire underwent translation into Malagasy by a local translator proficient in English. Subsequently, back-translation ensured the accuracy of translation and preservation of information integrity. Before its implementation, ten mothers from the health center reviewed the questionnaire and provided feedback. Based on the feedback, the number of questions was reduced due to the observed decrease in the women’s attention span after 25 min. Mothers who reviewed the questionnaire were excluded from the study to prevent bias. At the conclusion of the questionnaire administration, anthropometric measurements of both the mother and child were recorded, and the mother’s clinical history was consulted if available.

The anthropometric assessments were always carried out by the same experienced health workers, who have extensive training in anthropometric measurements and seven years’ experience in the Change Onlus Child Malnutrition Prevention and Treatment Project. However, no additional inter-rater reliability checks were performed.

For maternal measurements, an SECA stadiometer captured height to the nearest 0.1 cm, while an electronic scale (SECA Clara 803, Hamburg, Germany) recorded weight to the nearest 0.1 kg. Two measurements were taken for the mother’s height, and an average was calculated to minimize the measurement error. The mother’s BMI was then calculated by dividing weight by the square of height (kg/m^2^), and their nutritional status was categorized as underweight, normal, overweight, or obese based on the WHO cut-off values.

A UNICEF wooden measuring board was used to measure length in the prone position to an accuracy of ±0.2 cm. The positioning of the child during the measurements was supervised by the health worker, with the assistance of the child’s mother and a nutritionist employed by the NGO, to ensure the reliability of the measurements.

All raw anthropometric data were entered into the WHO Anthroplus software to calculate length/height-for-age (HFA), weight-for-age (WFA), and body mass index-for-age (BAZ), while an online tool was used to calculate weight-for-height (WFH).

The anthropometric status was determined using cut-off points according to the criteria established by the WHO [21]. For this study, stunting was defined as height or length at or below −2 standard deviations (SDs) from the median for the same age and sex (HFA z-score ≤ −2). Meanwhile, wasting was defined as weight at or below −2 SDs from the median for the same length/height and sex (WFH z-score ≤ −2). Other classifications using other anthropometric values following WHO Child Growth Standards are also presented, but all analyses have been performed with the stunting and wasting definitions mentioned above.

### 2.5. Data Analysis

Mother and child characteristics were analyzed with descriptive statistics and presented as means and SDs, medians, and interquartile ranges or as frequencies and percentages. The normality of the distribution was determined using the Kolmogrov–Smirnov test. The comparisons of variables between stunted children vs non-stunted children were made using the Chi-square test or Mac Nemar test for qualitative variables and the ANOVA test. Statistical significance was set at α < 0.05. Prevalence ratios (PR) with 95% confidence intervals (95% CIs) were obtained based on unconditional logistic regression. An adjusted multivariate regression model was also tested, and the results are presented; however, the wide 95% CIs obtained suggest that the results may not be sufficiently reliable, possibly due to the limited sample size. The Cohen’s kappa coefficient (indicating agreement) was determined for each real WFH classification against the theoretical WFH classification. All analysis were performed using IBM SPSS Statistics version 26.0 (IBM SPSS Statistics, Chicago, IL, USA)

## 3. Results

In this study, 437 mother–child pairs participated. Of the children, 251 (57.4%) had an HFA z-score ≤ −2 and were classified as stunted, and 186 (42.6%) had an HFA z-score > −2 and were classified as non-stunted.

### 3.1. General Characteristic of the Mothers

The general profile of the mothers is shown in Table 1. Results revealed statistically significant differences between the stunted and non-stunted groups regarding the maternal weight, height, and BMI. This indicates a close correlation between maternal and child anthropometry.

A significant difference appeared in birth spacing under 24 months, which was present in 7.9% of stunting cases and 3.2% of non-stunting cases. Mothers with a lower level of education (primary or below) represented 51.2% of mothers of stunted children and 37.3% of mothers of non-stunted children, with a significant difference between the two groups.

### 3.2. Profile of the Infants

The general characteristics of the infants are shown in Table 2. The incidence rate of stunting increased with age from 24.2% in the 0–6-months age group to 33.7% in the 7–13-months age group and further to 42.1% in the 14–24-months age group with a significant difference across different age groups. All anthropometric parameters exhibited a statistically significant difference between the two groups. The mid-upper arm circumference (MUAC), measured only in children aged ≥6 months (n = 346), revealed that 4.3% of stunted children had an MUAC ≤ 114 mm (severe wasting), and 26.1% had an MUAC ≥ 115 mm–≤ 124 mm (moderate wasting). The non-stunted group exhibited significantly different MUAC values, with no children with an MUAC ≤ 114 mm (severe wasting) and only 8.9% with an MUAC ≥ 115 mm–≤ 124 mm (moderate wasting). Contrary to the expectations of this study, the total incidence rate of wasting was approximately 12.4%. Only 3% of children had severe wasting, and 9.4% moderate wasting.

The mean SD for WFA values was −2.07 ± 1.33 in the stunted group and −0.65 ± 1.08 in the non-stunted group. The SD values for this parameter, which defines “underweight”, demonstrated a significant difference between the two groups.

HFA values provided more detailed insight into the phenomenon of stunting and is the variable that was used for stunting classification in this study. Among the 251 instances of stunting identified, 129 (51.4%) exhibited HFA indices with an SD value indicative of severe stunting and 122 (48.6%) exhibited HFA indices with an SD value suggestive of moderate stunting.

### 3.3. Maternal Care and Feeding Practices

The maternal care and feeding practices of mothers and children are shown in Table 3. Only 83.8% of the women took iron and folic acid supplements during pregnancy. The most common method of delivery was vaginal, accounting for 92.0% of births, and the type of delivery showed a statistically significant difference between the two groups.

A total of 35.9% of the sample gave birth at home, and only 57.4% of the mothers initiated breastfeeding within the first hour after delivery. Those who initiated breastfeeding in the first hour represented 55.8% of the total number of women with stunted children and 59.7% of those with non-stunted children.

Among the 304 children aged one year or older included in the study, 96.1% continued to receive breastfeeding until 12 months. There was a statistically significant difference between the two groups: 99.1% of non-stunted children continued breastfeeding until 12 months, while the percentage dropped to 94.4% in stunted children.

### 3.4. Socio-Economic Conditions of the Household

The socio-economic characteristics of the household are described in Table 4. The interviewed mothers had an average family size of 4.59 ± 1.69 individuals. The overcrowding rate m^2^/person indicates that 51.4% of children affected by stunting lived in overcrowded conditions (average < 5 m^2^/person) [22,23], and the percentage dropped to 44.1% in the non-stunted group.

In 75.1% of cases, the interviewed women had an average family income of less than 200,000 MGA Ariary (~40 €). Mothers with a monthly income exceeding 200,000 MGA Ariary represented only 21.5% of the total stunted group.

Rice, a staple of the traditional Malagasy diet, was available for less than six months for 32% of the women, representing 34.7% of the stunted group and 28.5% of the non-stunted group.

### 3.5. Infant Anthropometry and Overlapping Nutritional Conditions

A cross-tabulation of different overlapping nutritional conditions is presented in Table 5. When simultaneously studying WFH and stunting, most children fell within the “Normal WFH” and “Not stunted” category. However, those affected by moderate or severe stunting were also more likely to fall into the moderate or severe wasting categories. Within the “Not stunted” group, 11.8% of children were wasted while 84.5% of non-wasted children were stunted.

**Figure 1:**
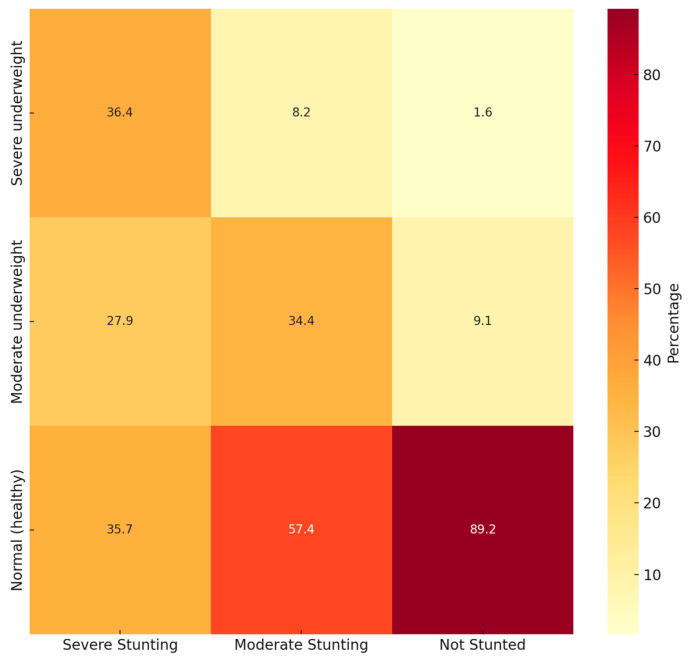
Heatmap of simultaneous WFA and stunting status.

**Figure 2:**
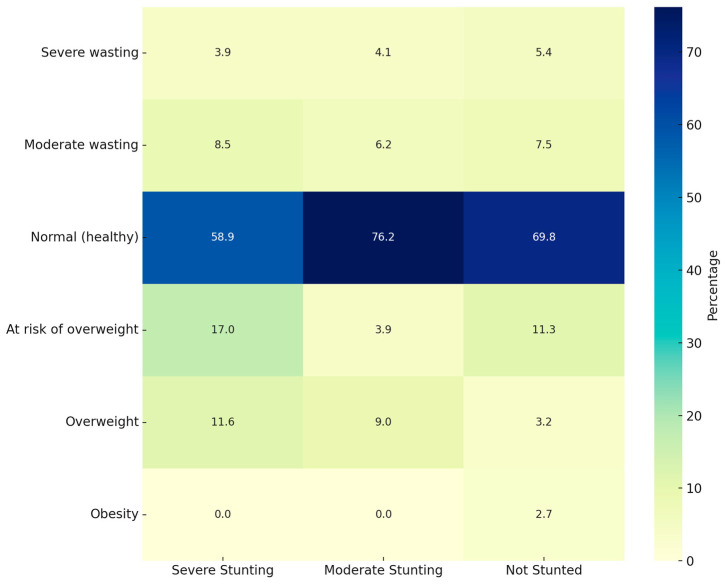
Heatmap of simultaneous BAZ and stunting status.

Figure 1 is a heatmap for the simultaneous WFA and stunting classification and Figure 2 for the simultaneous BAZ and stunting classification.

When studying WFA and stunting simultaneously, most children fell within the “Normal WFA” and “Not stunted” category. However, 57.4% of “Moderately stunted” children also presented “Normal WFA”. Something similar appears when studying BAZ and stunting simultaneously, with most children in the “Normal BAZ” category but with a high prevalence of “Moderately stunted” children.

### 3.6. Infant Anthropometry and Impact of Wasting

Table 6 presents the results of the study regarding the impact of wasting in the study population. In the first section (Table 6(a)) labeled “Real child height”, data related to the parameter WFH obtained using the real weight and height of the children are presented. In the second section (Table 6(b)) labeled “Adjusted to WHO height for age”, data related to the parameter WFH obtained using the theoretically expected height of the children are presented. This latter data are the results of a preliminary speculative analysis using the expected height-for-age according to the WHO HFA standards instead of the real observed height of the children.

The mean difference between the real height and the expected height of children in the study should have been based on their age and was −5.23 ± 5.51 cm, with a mean of −7.73 ± 3.19 cm in the stunted group and of −1.60 ± 3.34 cm in the not stunted group. The difference between the actual and expected height showed a statistically significant difference between the two groups.

The results obtained from actual anthropometric data showed that only 3% of the total children were affected by severe wasting, and only 9.4% were affected by moderate wasting. Malnutrition in terms of WFH parameters has a relatively low impact, with an average incidence rate of 12.4%. However, adjusting the values using the expected HFA, according to the WHO standards, significantly changed the estimated incidence of wasting.

### 3.7. Theoretical Impact of Wasting

The data presented in Table 7 provide insight into the actual incidence of wasting among the studied population, along with an estimated incidence if the children were to have an appropriate height for their age, according to the standards set forth by the WHO.

Upon examination of these preliminary and speculative data, it is observed that when children’s height is brought in line with the WHO standards for their age, 27.2% (n = 119) of the sample would be affected by severe wasting. Of these, using actual weight and height parameters, only 2.3% (n = 10) would be identified as severely wasted.

Using actual weight and height parameters, only seven cases of moderate wasting can be identified, with an incidence rate of 1.6%. However, if the children’s heights were appropriate for their age according to WHO standards, the incidence rate would increase to 15.1%, or 66 cases.

Following the same logic, we see that only 32.2% of the normal cases would match if the height was adjusted to the height-for-age standards proposed by the WHO.

The result of the Cohen’s kappa analysis is κ 0.076 (standard error 0.027; approximate T 3.29; approximate significance <0.001), indicating a low level of agreement between the observed and predicted rates of malnutrition. This suggests that the incidence of diagnosed wasting may have been underestimated, particularly in cases of severe malnourishment. Additionally, the results suggest an overestimation of the rates of overweight and obesity.

However, it must be noted that no alternative classification adjustments or sensitivity analyses were conducted and the analysis for the determination of the estimated theoretical prevalence of wasting using the expected HFA values determined by the WHO is preliminary and speculative and should not be interpreted to reflect the actual state of the children.

## 4. Discussion

The children included in this study present a stunting prevalence rate of 57.4%, a rate that is higher than the most recent national data from 2021, which showed a rate of 35.5% for children under two [24]. The determinants of the incidence of stunting were similar to those of other studies and included maternal anthropometric parameters [25,26] and the level of education [25,27,28,29].

Stunting has adverse effects on development and is a well-established risk indicator for poor child outcomes. Diagnosis before the age of two is a predictor of delayed cognitive development and poorer educational outcomes in childhood and adolescence [30,31,32]. It is correlated with lower economic development, with short and long-term consequences for the individual, the family unit, and the community as a whole [30,31,32]. Breastfeeding, when continued for at least the first year of the child’s life, has been shown to be a protective factor [33].

Pregnancy and the postnatal period are the periods of most rapid brain development. The cerebral cortex and neural tube begin to form approximately 22 days after conception, and adequate nutrition is essential from the beginning of pregnancy [30]. Children who have suffered from stunting in the first two years of life do not score in the normal range on intelligence quotient (IQ) tests at a school age when compared to children of the same age who had not suffered from malnutrition [30]. Similar patterns may be repeated in cases of severe wasting; children who have experienced episodes of severe wasting in their early years have lower IQs compared to their siblings who have not experienced episodes of malnutrition and also reported greater behavioral problems [32].

The impact of stunting on fat reserves remains uncertain, but a reduction in skinfold thickness or body fat has been reported in stunted children [34,35]. However, stunted children may, at the same time, be classified as overweight using WFH values, as seen in this study. This might suggest that these stunted children may also have excess body fat [36]. As indicated in several studies, fat reserves are profoundly depressed in cases of wasting [1] Additionally, the significant reduction in muscle mass seen with wasting in comparison to stunting may explain the higher risk of death associated with wasting [9].

This study also focuses on the possibility that, in low-resource settings where stunting is prevalent, the identification of wasting may be underestimated. The aim of the preliminary and speculative analysis performed in this study was to calculate the WFH value using the expected height that children should have reached based on their age in order to ascertain whether the high incidence of stunting might have represented a limitation in the diagnosis of wasting. The results of this speculative analysis led to the proposed idea that if the children in the study had a height appropriate for their age and maintained their current real weight, 42.3% (n = 185) of the total cases would then be classified as suffering from wasting instead of the 12.4% classified as wasted when using the real height values.

In general, wasting in children under two years old can be frequently underestimated due to a combination of methodological, biological, and programmatic factors. The underestimation of wasting prevalence among children under two years old in rural Madagascar can be attributed to several interrelated factors.

The most common sources of misclassification are errors in the anthropometric measurements used for assessing the nutritional status of children, particularly in the early years of life. Anthropometric measurements, particularly length, height, and MUAC, are prone to errors due to the challenges of measuring young children [37,38,39]. The training and skills of measurers also play a crucial role in reducing anthropometric measurement errors. This underscores the importance of standardized techniques and regular training for measurers.

Digit preference, where measurers tend to round measurements to certain numbers, is another common source of error [39,40]. Data recording errors can lead to implausible values, which are measurements that fall outside the biologically possible range. These errors can significantly affect the prevalence estimates of malnutrition and stunting. Quality control and data validation are critical for ensuring the accuracy of anthropometric data.

Another reason for the underestimation is the reliance on point-prevalence data, which do not capture the high incidence of transient wasting episodes typical in this age group. Thus, many cases may go undetected unless longitudinal monitoring is conducted [41]. For example, seasonal variations significantly impact food security. During lean periods, food scarcity intensifies, leading to higher wasting rates that may not be captured in surveys conducted during post-harvest seasons.

Additionally, structural issues, like limited access to healthcare facilities, especially in rural or marginalized communities, hamper the regular monitoring and timely detection of malnutrition cases, resulting in underreporting [42]. Socioeconomic challenges, including poverty and low maternal education levels, further exacerbate the situation by restricting access to nutritious food and diminishing awareness of the early signs of malnutrition and care-seeking, further skewing the data.

These factors collectively may lead to a significant underestimation of the true prevalence of wasting among young children in these communities. The misclassification of children can lead to incorrect estimates of malnutrition prevalence, which can, in turn, affect policy decisions and resource allocation. These misestimations are critical because interventions during the “first 1000 days” are most effective, and failure to accurately assess the prevalence undermines the scale and urgency of needed responses. Improved data collection and analysis methods and targeted outreach may significantly enhance the detection and treatment of malnutrition in this vulnerable age group in similar low-resource settings.

Global health policies categorize malnutrition in children under two years of age as a critical concern, recognizing its multifaceted nature, due to its irreversible impact on physical and cognitive development if not addressed promptly. In terms of treatment and strategies, global health policies include several key components: assessment and surveillance, integrated and multisectoral approaches, micronutrient supplementation and food fortification, treatment protocols for severe wasting, a focus on maternal and child health, and addressing the double burden of malnutrition.

The WHO has proposed a number of solutions to reduce the incidence of stunting globally, including (i) maternal, infant, and young child nutrition and micronutrient supplementation through community and health interventions to improve maternal nutrition and infant and young child feeding practices, (ii) the management of severe wasting and nutrition in emergency situations through early identification and the treatment of wasting cases, and iii) improved water and sanitation in communities [43].

The WHO and UNICEF emphasize a “window of opportunity” from conception to 24 months, advocating for interventions such as exclusive breastfeeding for the first six months, timely introduction of complementary foods, and continued breastfeeding up to two years or beyond [44]. International frameworks like the “Ten Steps to Successful Breastfeeding” and the Global Strategy for Infant and Young Child Feeding shape national strategies and guide policymakers in creating health systems that support early nutrition interventions [45]. Moreover, the Sustainable Development Goal 2 calls for ending all forms of malnutrition by 2030, encouraging context-specific, multi-sectoral approaches that involve local governments, NGOs, and international bodies to address undernutrition and food insecurity [46]. This comprehensive and coordinated framework, encompassing assessment, integrated treatment, targeted supplementation, and multisectoral collaboration, forms the backbone of global strategies to both classify and treat malnutrition effectively [47] and ensures that guidelines remain evidence-based and are continuously refined based on emerging data and the changing epidemiologic landscape [47,48,49].

Overall, global health policies are designed to be responsive to local needs while staying grounded in internationally recognized standards. Despite these efforts, a gap remains in globally standardized policies for disease-related malnutrition, with limited integration into broader health policies, especially in low-resource settings [50]. Thus, while the policy landscape is shaped by strong global guidelines, successful implementation hinges on tailored national strategies and sustained political will [39].

The most recent document released by the WHO in November 2023 [51] for the prevention and treatment of wasting states that nutritional treatment by means of therapeutic feeding is only indicated for patients diagnosed with moderate and/or severe wasting and/or nutritional edema. This could result in a lack of access to the therapeutic foods necessary for weight and height recovery, thereby affecting overall survival, health, and development.

The term “nutritional treatment” is used in this context to describe the provision of regular outpatient services and potentially hospital services (if required) through which children with severe wasting and/or nutritional edema receive therapeutic milk or ready-to-use therapeutic foods with the objective of achieving anthropometric recovery and resolving the edema. Despite the lack of evidence that therapeutic foods are a solution for treating growth arrest, short-term nutritional improvements during fetal life and childhood can lead to an average increase in height of up to 8 cm compared to the parents’ average height [52].

There is ample evidence that when children with wasting are fed an energy- and nutrient-rich diet and any infections are treated at the same time, weight recovery occurs at rates up to 20 times higher than normal weight gain [53]. Although treated children show a good response in terms of weight gain, there is no significant change in height gain in treated cases [7].

Stunting may be untreatable and require preventive measures [54]. However, it remains unclear which strategies are most effective at the community level and at what time they can make a difference [10,55,56]. It can be stated with certainty that stunting represents a form of malnutrition with irreversible effects, the severity of which may be exacerbated when combined with untreated wasting through therapeutic feeding [12,57].

The application of these guidelines to our sample indicates that 76 of the 437 (17.4%) children would have been eligible for community therapeutic feeding services on the basis of an MUAC value < 125 mm. Upon consideration of the real parameters of WFH as recorded in the field, only 12.4% of the total cases (3% severe, 9,4% moderate) would have had access to therapeutic food-distribution community services for the treatment of wasting according to the parameters established by the WHO. Furthermore, the results from the preliminary speculative analysis using the expected height suggest that up to 42.3% of the total cases might benefit from therapeutic feeding targeted to tackle wasting. This difference could mean that children suffering from wasting may not be able to access treatment due to their stunting, which in itself is a marker of malnutrition.

Wasting and stunting are often presented as two distinct forms of malnutrition that require different interventions for prevention and/or treatment. Nevertheless, it is evident that these two forms of malnutrition are closely related and frequently occur concurrently. In the studied population, 24.9% of the severely stunted children and 11.5% of moderately stunted children suffer from wasting. Meanwhile, 81.5% of the severely wasted children and 58.5% of moderately wasted children suffer from stunting.

Wasting and stunting are both associated with increased mortality, particularly when present in the same child [10,58,59]. The irreversible effects of stunting and the negative effects of the simultaneous existence of wasting and stunting make it imperative to assess how well wasting treatment is provided at the community level in contexts where stunting has such a high incidence rate and how useful it is to separate the two forms of malnutrition.

In order to correctly assess the prevalence of acute malnutrition/wasting through the anthropometric status of a child in an area of low economic resources where chronic malnutrition/stunting may be prevalent, it would be advisable to first assess height for stunting before weight-for-height ratio parameters to assess wasting and that it may be advisable to use not the real height of the child but the expected for their age. This could identify children with chronic malnutrition/stunting that are not classified as having acute malnutrition/wasting but that could still benefit from nutritional interventions. This highlights the importance of considering the mean height-for-age as a reference in the interpretation of nutritional indicators in similar contexts, to ensure the more accurate detection of cases in need of intervention.

### Strengths/Limitations

This study is strengthened by its well-organized and monitored randomized data collection. Systematic random sampling was used to choose the women invited to participate, which should ensure the representativeness of the sample. However, selection bias may still exist as the original list from which participants were selected to be invited comes from a user registry of the health center.

Furthermore, to the best of our knowledge, this study is the first to evaluate the possibility of underestimating wasting in settings with a high prevalence of stunting.

It should be noted that the study’s findings may not be very broadly generalizable given the very specific study population and setting; nevertheless, the findings may be generalized to similar impoverished contexts, and this study could represent a replicable model in other rural areas, with similar demographic situations, such as a low population density, where epidemiological investigations cannot be carried out on a large scale in health centers but in homes, and with the limitations noted here for investigating aspects related to public health and prevention. This situation, although not generalizable to the entire continent, is representative of many rural regions in Africa and in other low-socioeconomic-resource contexts that impact nutrition and can affect the anthropometric development of children, so that the results could contribute to the design of similar interventions in comparable areas.

However, applying findings from a study conducted in rural Madagascar to broader populations necessitates the careful consideration of regional similarities and differences in health systems, disease prevalences, and socio-economic contexts. The geographical and socio-economic similarities among rural populations in Sub-Saharan Africa suggest that health-related findings from rural Madagascar underscore the universal challenges of access, infrastructure, and nutrition that resonate throughout Sub-Saharan Africa. When it comes to stunting and wasting rates, a recent multi-country analysis from Demographic and Health Survey data across 34 Sub-Saharan African countries revealed that approximately 34.04% of under-five children are stunted, with national rates ranging from 16.14% in Gabon to 56.17% in Burundi, and that the most affected age bracket is often under 24 months [60]. Similarly, the prevalence of wasting in the region stands at 7.09% overall, with 4.97% moderately and 2.12% severely wasted, with evidence showing that children under two bare a disproportionate share of this burden [61]. Given the similar context, successful strategies implemented in Madagascar may potentially be adapted and applied elsewhere, promoting the overall enhancement of maternal and child health services across diverse sub-Saharan settings.

The primary limitations of this study are the possible errors related to the anthropometric measurements and the use of the theoretical calculation of WFL values, derived by adjusting the height of the children in our sample to the WHO international HFA standards. Errors in anthropometric measurements among children under two are indeed common, primarily due to the challenges associated with accurately measuring infants and toddlers, such as their inability to remain still and the difficulty in positioning them correctly.

The prevalence of wasting derived from the simulated data does not represent the clinical reality, and the use of such speculative estimates must be clearly separated from empirical findings and should be interpreted as an exploratory and speculative analysis with caveats and should not form the main basis of the conclusions. This approach does not provide an accurate estimation of the number of children who do not receive treatment for wasting at the community level.

## 5. Conclusions

The findings of this study indicate that stunting affects over half of the studied pediatric population, while the rate of wasting was under the expected value at around 1 out of every 8 of the children studied. An untested speculative analysis, adjusting height to the WHO HFA standard, estimated that the prevalence of wasting could be as high as 1 out of 2.5 children, with many cases possibly exhibiting severe wasting.

This suggests that wasting and stunting should perhaps be considered as separate but interconnected forms of malnutrition that require different assessments and coordinated interventions for prevention and/or treatment. Community-level nutritional screening should include separate assessments for both types of malnutrition, stunting and wasting, and interventions should be tailored to the specific nutritional condition(s) presented by each child.

In populations presenting a known significant prevalence of stunting, specific care in the adequate assessment of wasting, perhaps using several different assessment methods or variables, must be taken in order to make sure that children suffering from wasting that may be somewhat masked by their stunting are not excluded from interventions from which they may greatly benefit. Measuring the nutritional status using a single index can make it difficult to correctly assess the different forms of malnutrition as there may be overlaps, and therefore, this may not reflect the anthropometric failure rate in a population.

The use of indicators such as MUAC or MUAC-for-age, which are less height-dependent, is essential for accurate assessments in stunted populations. Combining these with WFA could result in diagnostic accuracy and help identify high-risk children who may be missed by WFH or BAZ-based criteria alone. The Composite Index of Anthropometric Failure (CIAF), which combines three anthropometric indices, WFA, HFA, and WFH, and extended CIAF (ECIAF), which combines the characterization of malnutrition due to undernutrition and excess weight, could also be useful tools for classifying children with multiple nutritional deficits like concurrent wasting and stunting.

Further research is still needed to examine the interactions between different forms of malnutrition in order to understand the complex relationship between stunting and wasting and develop effective solutions targeting both in low-resource settings.

## Figures and Tables

**Table 1:** General characteristics of the mothers by stunting classification of their children.

	Total (n = 437) Frequency (%) Mean ± SD	Stunted (n = 251) Frequency (%) Mean ± SD	Not Stunted (n = 186) Frequency (%) Mean ± SD	*p*-Value ^1^	PR	95% CI	aPR ^2^	a95% CI ^2^
Age	25.8 ± 6.2	25.7 ± 6.4	25.89 ± 5.8	0.769				
<18	31 (7.1%)	20 (7.9%)	11 (5.9%)	0.524	0.90	0.67–3.14	1.74	0.29–10.40
18–29	297 (68%)	165 (65.5%)	132 (71.4%)		1 Reference		1 Reference	
30–39	90 (20.6%)	54 (21.4%)	36 (19.5%)		0.55	0.44–1.94	1.64	0.90–2.99
40–49	19 (4.3%)	13 (5.2%)	6 (3.2%)		0.50	0.44–4.68	1.50	0.52–4.38
Weight (kg)	48.6 ± 8.0	46.9 ± 6.8	50.9 ± 9.0	<0.001				
Height (cm)	152.7 ± 5.7	151.7 ± 5.4	153.9 ± 5.8	<0.001				
BMI (kg/m^2^)	20.8 ± 3.0	20.3 ± 2.7	21.4 ± 3.4	<0.001				
<18.5	108 (24.7%)	63 (25%)	45 (24.3%)	0.015	0.91	0.58–1.43	0.37	0.16–0.86
18.5–24.9	289 (66.1%)	175 (69.4%)	114 (61.6%)		1 Reference		1 Reference	
25.0–29.9	34 (7.8%)	13 (5.2%)	21 (11.4%)		0.40	0.19–0.84	3.18	0.90–11.17
>30	6 (1.4%)	1 (0.4%)	5 (2.7%)		0.13	0.02–1.13	4.97	0.31–79.71
Pathologies (no)	420 (96.1%)	239 (95.2%)	181 (97.3%)	0.263				
Health status				0.273				
Healthy	420 (96.1%)	239 (95.2%)	181 (97.3%)		1 Reference		1 Reference	
Any illness	17 (3.9%)	12 (4.8%)	5 (2.7%)		1.82	0.63–5.25	1.19	0.36–3.99
Acute	1 (0.2%)	1 (33.3%)	0 (0.0%)		-	-	-	-
Chronic	5 (1.1%)	2 (66.7%)	3 (100%)		-	-	-	-
Parity				0.721				
Primiparous	161 (36.8%)	95 (37.7%)	66 (35.7%)		1 Reference		1 Reference	
2–3	203 (46.5%)	113 (44.8%)	90 (48.6%)		0.87	0.57–1.33	0.36	0.10–1.46
≥4	73 (16.7%)	44 (17.5%)	29 (15.7%)		1.05	0.60–1.85	0.33	0.08–1.34
Twin pregnancy ^3^	13 (3.0%)	9 (3.6%)	4 (2.2%)	0.392	1.67	0.51–5.53	0.72	0.43–1.20
Birth spacing < 24 months	26 (5.9%)	20 (7.9%)	6 (3.2%)	0.040	2.57	1.01–6.53		
Education				0.025				
Illiterate	17 (3.9%)	12 (4.8%)	5 (2.7%)		2.86	0.87–9.43	0.51	0.12–2.24
Primary	181 (41.4%)	117 (46.4%)	64 (34.6%)		2.17	1.13–4.19	0.37	0.10–1.32
Secondary 1st cycle	193 (44.2%)	102 (40.5%)	91 (49.2%)		1.33	0.7–2.54	0.60	0.17–2.19
Secondary 2nd cycle	46 (10.5%)	21 (8.3%)	25 (13.5%)		1 Reference		1 Reference	
Education level				0.004				
Primary or below	198 (45.3%)	129 (51.2%)	69 (37.3%)		1.76	1.20–2.60		
Secondary or above	239 (54.7%)	123 (48.8%)	116 (62.7%)		1 Reference		1 Reference	
Occupation				0.165				
Farmer	335 (76.7%)	203 (80.6%)	132 (71.4%)		1 Reference		1 Reference	
Seller	41 (9.4%)	19 (7.5%)	22 (11.9%)		0.56	0.29–1.08	1.18	0.46–3.04
Fisher	26 (5.9%)	15 (6.0%)	11 (5.9%)		0.89	0.39–1.99	0.17	0.02–1.76
Housewife	5 (1.1%)	2 (0.8%)	3 (1.6%)		0.43	0.07–2.62	0.24	0.20–2.91
Other	30 (6.9%)	13 (5.2%)	17 (9.2%)		0.50	0.23–1.05	1.87	0.50–6.93

CI: confidence interval; PR: prevalence ratio; SD: standard deviation; ^1^ *p*-Value was obtained and calculated using ANOVA or a Chi-squared test; ^2^ adjusted for weight, height, BMI, birth spacing, and educational level; ^3^ twin pregnancy at any time during the mother’s lifetime.

**Table 2:** Profile of the infant by stunting classification.

	Total (n = 437) Frequency (%) Mean ± SD	Stunted (n = 251) Frequency (%) Mean ± SD	Not Stunted (n = 186) Frequency (%) Mean ± SD	*p*-Value ^1^	PR	95% CI	aPR ^2^	a95% CI ^2^
Age	10.5 ± 6.5	11.6 ± 6.3	9.2 ± 6.4	<0.001				
0–6 months	137 (31.4%)	61 (24.2%)	76 (41.1%)	<0.001	1 Reference		1 Reference	
7–13 months	148 (33.9%)	85 (33.7%)	63 (34.1%)		1.68	1.05–2.68	1.35	0.63–2.90
14–24 months	152 (34.8%)	106 (42.1%)	46 (24.9%)		1.87	1.77–4.65	2.40	1.10–5.20
Sex								
Female	217 (49.7%)	119 (47.2%)	98 (53.0%)	0.235	0.79	0.54–1.16	0.92	0.55–1.52
Male	220 (50.3%)	133 (52.8%)	87 (47.0%)		1 Reference		1 Reference	
Low birth weight ^3^								
Yes	348 (79.6%)	185 (73.7%)	163 (87.6%)	<0.001	0.39	0.23–0.66	1.57	0.80–3.08
No	89 (20.4)	66 (26.3%)	23 (12.4%)		1 Reference		1 Reference	
Weight (kg)	7.3 ± 1.7	7.2 ± 1.6	7.5 ± 1.9	0.033				
Height (cm)	66.2 ± 8.5	65.2 ± 7.9	67.6 ± 9.1	0.003				
MUAC (mm) (n = 346)	135.9 ± 11.7	133.0 ± 11.8	140.4 ± 11.3	<0.001				
≤114 mm Severe wasting	9 (2.6%)	9 (4.3%)	0 (0%)	<0.001	-	-	-	-
≥115 mm–≤124 mm Moderate wasting	67 (19.4%)	55 (26.1%)	12 (8.9%)		3.83	2.96–7.48	1.0	0.43–2.34
≥125 mm Normal	270 (78.0%)	147 (69.7%)	123 (91.1%)		1 Reference		1 Reference	
Weight-for-height (WFH)	−0.01 ± 1.85	−0.09 ± 2.01	−0.16 ± 1.60	0.157				
<−3 SD Severe wasting	13 (3.0%)	8 (3.2%)	5 (2.7%)	0.135	1.28	0.41–4.02	0.05	0.1–0.19
≥−3 to ≤−2 SD Moderate wasting	41 (9.4%)	24 (9.5%)	17 (9.2%)		1.13	0.58–2.20	0.006	0.01–0.05
>−2 to ≤+1 SD Normal	283 (64.8%)	157 (62.3%)	126 (68.1%)		1 Reference		1 Reference	
>+1 to ≤+2 SD Overweight risk	42 (9.6%)	22 (8.7%)	20 (10.8%)		0.88	0.46–1.69	2.5	1.01–5.94
≥+2–≤+3 SD Overweight	26 (5.9%)	15 (6.0%)	11 (5.9%)		1.09	0.48–2.46	4.51	1.07–18.92
>+3 SD Obesity	32 (7.3%)	26 (10.3%)	6 (3.2%)		2.86	1.20–6.84	-	-
Weight-for-age (WFA)	−1.5 ± 1.4	−2.1 ± 1.3	−0.7 ± 1.1	<0.001				
<−3 SD Severe underweight	60 (13.7%)	57 (22.6%)	3 (1.6%)	<0.001	6.56	3.69–11.67	18.58	4.82–71.58
≥−3 to ≤−2 SD Moderate underweight	95 (21.7%)	78 (31.0%)	17 (9.2%)		27.19	8.31–88.92	6.51	3.27–12.94
≥−1 SD Normal	282 (64.5%)	117 (46.4%)	165 (89.2%)		1 Reference		1 Reference	
Height-for-age (HFA)	−2.2 ± 1.8	−3.3 ± 1.1	−0.6 ± 1.3	<0.001				
<−3 SD Severe stunting	129 (29.5%)	129 (100%)	0 (0%)	<0.001	-	-	-	-
≥−3 to ≤−2 SD Moderate stunting	122 (27.9%)	122 (48.6%)	0 (0%)		-	-	-	-
≥−1 SD Normal	186 (42.6%)	0(0%)	186 (100%)		-	-	-	-
BMI for Age (BAZ)	−0.2 ± 1.7	−0.1 ± 1.8	−0.4 ± 1.5	0.064				
<−3 SD Severe wasting	20 (4.6%)	10 (4.0%)	10 (5.4%)	0.034	0.77	0.31–1.90	2.81	0.84–9.40
≥−3 to ≤−2 SD Moderate wasting	33 (7.6%)	19 (7.5%)	14 (7.6%)		1.04	0.54–2.16	1.19	0.55–2.60
≥−2 to ≤+1 SD Normal	299 (68.4%)	169 (67.1%)	130 (70.3%)		1 Reference		1 Reference	
>+1 to ≤+2 SD Overweight risk	49 (11.2%)	28 (11.1%)	21 (11.4%)		1.02	0.55–1.88	0.24	0.08–0.71
≥+2–≤+3 SD Overweight	31 (8.2%)	26 (10.3%)	6 (3.2%)		1.75	0.83–3.68	0.09	0.2–0.35
>+3 SD Obesity	5 (1.1%)	0 (0%)	5 (2.7%)		-	-		

CI: confidence interval; MUAC: middle upper arm circumference; PR: prevalence ratio; SD: standard deviation; ^1^ *p*-Value was obtained and calculated using ANOVA or a Chi-squared test; ^2^ adjusted for age, low birth weight, MUAC, weight-for-age and sex; ^3^ low birth weight was considered as <2.5 kg.

**Table 3:** Maternal care and feeding practices by stunting classification of their children.

	Total (n = 437) Frequency (%) Mean ± SD	Stunted (n = 251) Frequency (%) Mean ± SD	Not Stunted (n = 186) Frequency (%) Mean ± SD	p-Value ^1^	PR	95% CI	aPR ^2^	a95% CI ^2^
ANC ^2^								
0	3 (0.7%)	2 (0.8%)	1 (0.5%)	0.529	1.55	0.14–17.25	17.72	0.01–177.24
1	7 (1.6%)	5 (2%)	2 (1.1%)		1.94	0.37–10.12	3.27	0.21–51.03
2–3	31 (7.1%)	21 (8.4%)	10 (5.4%)		1.63	0.75–3.50	1.72	0.28–23.19
≥4	396 (90.6%)	223 (88.8%)	173 (93.0%)		1 Reference		1 Reference	
IFA supplementation								
Yes	366 (83.8%)	209 (83.3%)	157 (84.4%)	0.749	1 Reference		1 Reference	
No	71 (16.2%)	42 (16.7%)	29 (15.6%)		1.08	0.65–1.82	0.78	0.25–1.43
Type of delivery								
Vaginal	402 (92.0%)	239 (95.2%)	163 (87.6%)	0.013	1 Reference		1 Reference	
Operative vaginal birth	23 (5.3%)	7 (2.8%)	16 (8.6%)		0.30	0.12–0.74	0.52	0.17–1.58
Caesarean section	12 (2.7%)	5 (2.0%)	7 (3.8%)		0.49	0.15–1.56	0.62	0.15–2.55
Place of delivery								
Home	157 (35.9%)	97 (38.6%)	60 (32.3%)	0.169	1.32	0.88–1.97	0.17	1.43–0.86
Health center	280 (64.1%)	154 (61.4%)	126 (67.7%)		1 Reference		1 Reference	
Reason in case of home delivery								
Upcoming birth	72 (45.0%)	47 (47.5%)	25 (41.0%)	0.736	1 Reference		1 Reference	
Personal choice	60 (37.5%)	33 (33.3%)	27 (44.3%)		0.65	0.32–1.31	0.1	0.01–2.56
Transport issues	19 (11.9%)	12 (12.1%)	7 (11.5%)		0.91	0.32–2.60	1.0	0.1–13.25
Homecare for a matron	4 (2.5%)	3 (3.0%)	1 (1.6%)		1.60	0.16–16.15	0.1	0.01–45.02
Lack of money	4 (2.5%)	3 (3.0%)	1 (1.6%)		1.60	0.16–16.15	1.0	0.01–21.02
Absence of health staff	1 (0.6%)	1 (1.0%)	0 (0%)		-	-		
Early breastfeeding initiation								
Within the 1st hour after birth	251 (57.4%)	140 (55.8%)	111 (59.7%)	0.415	1 Reference		1 Reference	
After the 1st hour	186 (42.6%)	111 (44.2%)	75 (40.3%)		1.17	0.80–1.72	1.02	0.63–1.65
EBF (n = 385)								
Yes	170 (44.2%)	99 (42.9%)	71 (46.1%)	0.530	1 Reference		1 Reference	
No	215 (55.8%)	132 (57.1%)	83 (53.9%)		1.14	0.75–1.71	1.27	0.79–2.07
Breastfeeding up to 1-year (n = 304)								
Yes	292 (96.1%)	186 (94.4%)	106 (99.1%)	0.047	1 Reference		1 Reference	
No	12 (3.9%)	11 (5.6%)	1 (0.9%)		6.27	0.80–49.23	6.20	0.78–49.32
Weaning age (n = 368)								
Before 6 months	224 (60.9%)	142 (63.1%)	82 (57.3%)	0.269	1.27	0.83–1.95	6.02	0.76–47.47
After 6 months	144 (39.1%)	82 (57.3%)	61 (42.7%)		1 Reference		1 Reference	
Improved mother’s diet during pregnancy								
Yes	235 (53.8%)	131 (52.2%)	104 (55.9%)	0.440	1 Reference		1 Reference	
No	202 (46.2%)	120 (47.8%)	82 (44.1%)		1.16	0.80–1.70	0.85	0.55–1.31
Improved mother’s diet during breastfeeding								
Yes	182 (41.6%)	102 (40.6%)	80 (43.0%)	0.619	1 Reference		1 Reference	
No	255 (58.4%)	149 (59.4%)	106 (57.0%)		1.10	0.75–1.62	1.12	0.76–1.63
Use of iodized salt								
Yes	330 (75.5%)	190 (75.7%)	61 (24.3%)	0.918	1 Reference		1 Reference	
No	107 (24.5%)	140 (74.3%)	46 (24.7%)		0.97	0.63–1.52	0.95	0.61–1.48

ANC: antenatal care; CI: confidence interval; EBF: exclusive breastfeeding for the first 6 months; IFA: iron and folic acid supplementation during last pregnancy; PR: prevalence ratio; SD: standard deviation; ^1^ *p*-Value was obtained and calculated using ANOVA or a Chi-squared test; ^2^ adjusted for type of delivery, place of delivery, and breastfeeding up to 1-year.

**Table 4:** Socio-economic conditions of the mothers by stunting classification of their children.

	Total (n = 437) Frequency (%) Mean ± SD	Stunted (n = 251) Frequency (%) Mean ± SD	Not Stunted (n = 186) Frequency (%) Mean ± SD	*p*-Value ^1^	PR	95% CI	aPR ^2^	a95% CI ^2^
Family size	4.6 ± 1.7	4.6 ± 1.8	4.6 ± 1.6	0.985				
<4 persons	256 (58.6%)	152 (60.6%)	104 (55.9%)	0.330	1 Reference		1 Reference	
>4 persons	181 (41.4%)	99 (39.4%)	82 (44.1%)		0.83	0.56–1.21	0.99	0.88–1.12
Dimension of the house	28.8 ± 24.1	25.6 ± 23.0	30.4 ± 25.6	0.243				
<24	224 (51.3%)	140 (55.8%)	84 (45.2%)	0.028	1.53	1.04–2.24	1.0	1.01–1.05
>24	213 (48.7%)	111 (44.2%)	102 (54.8%)		1 Reference		1 Reference	
Overcrowding ^3^ (m^2^/person)	6.8 ± 6.5	6.5 ± 5.8	7.2 ± 7.40	0.260				
<5	211 (48.3%)	129 (51.4%)	82 (44.1%)	0.131	1.34	0.92–1.96	0.99	0.88–1.12
>5	226 (51.7%)	122 (48.6%)	104 (55.9%)		1 Reference		1 Reference	
Middle income ^4^								
<200,000 MGA Ariary	328 (75.1%)	197 (78.5%)	131 (70.4%)	0.054	1.53	0.99–2.37	0.69	0.45–1.07
≥200,000 MGA Ariary	109 (24.9%)	54 (21.5%)	55 (29.6%)		1 Reference		1 Reference	
Source of drinking water								
Public standpipe	149 (34.1%)	93 (37.1%)	56 (30.1%)	0.291	1 Reference		1 Reference	
Protected well	285 (65.2%)	156 (62.2%)	129 (69.4%)		0.73	0.49–1.09	1.47	0.97–2.23
Unprotected spring	3 (0.7%)	2 (0.8%)	1 (0.5%)		1.20	0.11–13.59	1.28	0.11–15.01
Rice availability								
<6 months	140 (32.0%)	87 (34.7%)	53 (28.5%)	0.172	1.33	0.88–2.00	0.99	0.88–1.11
≥6 months	297 (68.0%)	164 (65.3%)	133 (71.5%)		1 Reference		1 Reference	
Toilet facility								
Yes	398 (91.1%)	222 (88.4%)	94.6%)	0.025	1 Reference		1 Reference	
No	39 (8.9%)	29 (11.6%)	10 (5.4%)		2.30	1.09–4.84	2.015	1.01–4.58
Distance walking from water (min)	9.3 ± 11.3	10.1 ± 12.2	8.3 ± 9.7	0.098				
<5	270 (61.8%)	151 (60.2%)	119 (64.0%)	0.816	1 Reference		1 Reference	
>5	167 (38.2%)	100 (39.8%)	67 (36.0%)		1.18	0.80–1.74	0.99	0.88–1.11
Distance walking from health center (min)								
<45	233 (53.3%)	128 (51.0%)	105 (56.5%)	0.258	1 Reference		1 Reference	
>45	204 (46.7%)	123 (49.0%)	81 (43.5%)		1.25	0.85–1.82	1.03	0.99–1.09
Land ownership								
Yes	226 (51.7%)	132 (52.6%)	94 (50.5%)	0.671	1 Reference		1 Reference	
No	211 (48.3%)	119 (47.4%)	92 (49.5%)		0.92	0.63–1.35	0.85	0.57–1.25
Transport availability								
Yes	113 (25.9%)	57 (22.7%)	56 (30.1%)	1.46	1 Reference		1 Reference	
No	324 (74.1%)	56 (30.1%)	130 (69.9%)		1.46	0.95–2.25	1.34	0.86–2.07

CI: confidence interval; PR: prevalence ratio; SD: standard deviation; ^1^ *p*-Value was obtained and calculated using ANOVA or a Chi-squared test; ^2^ adjusted for dimension of the house, toilet facility, middle income, overcrowding, and rice availability; ^3^ overcrowding rate was considered as <5 m^2^ of floor area per person; ^4^ middle income was established in national currency of MGA Ariary.

**Table 5:** Overlapping nutritional conditions of the children.

	Severe Stunting <−3 SD (n = 129, 29.5%) Frequency (%)	Moderate Stunting ≥−3 to ≤−2 SD (n = 122, 27.9%) Frequency (%)	Not Stunted ≥−1 SD (n = 186, 42.6%) Frequency (%)	*p*-Value ^1^
Weight-for-height (WFH)				0.054
<−3 SD Severe wasting	18 (14.0%)	4 (3.3%)	5 (2.7%)	
≥−3 to ≤−2 SD Moderate wasting	14 (10.9%)	10 (8.2%)	17 (9.1%)	
>−2 to ≤+1 SD Normal	71 (55.0%)	86 (70.5%)	126 (67.7%)	
>+1 to ≤+2 SD Overweight risk	4 (3.1%)	11 (9.0%)	20 (10.8%)	
≥+2–≤+ 3 SD Overweight	11 (8.5%)	4 (3.3%)	11 (5.9%)	
>+3 SD Obesity	18 (14.0%)	7 (5.7%)	7 (3.8%)	
Weight-for-age (WFA)				0.001
<−3 SD Severe underweight	47 (36.4%)	10 (8.2%)	3 (1.6%)	
≥−3 to ≤−2 SD Moderate underweight	36 (27.9%)	42 (34.4%)	17 (9.1%)	
≥−1 SD Normal (healthy)	46 (35.7%)	70 (57.4%)	166 (89.2%)	
BMI for age (BAZ)				0.034
<−3 SD Severe wasting	5 (3.9%)	5 (4.1%)	10 (5.4%)	
≥−3 to ≤−2 SD Moderate wasting	11 (8.5%)	8 (6.2%)	14 (7.5%)	
>−2 to ≤+1 SD Normal (healthy)	76 (58.9%)	93 (76.2%)	130 (69.8%)	
>+1 to ≤+2 SD At risk of overweight	22 (17.0%)	5 (3.9%)	21 (11.3%)	
≥+2–≤+3 SD Overweight	15 (11.6%)	11 (9.0%)	6 (3.2%)	
>+3 SD Obesity	0 (0.0%)	0 (0.0%)	5 (2.7%)	

^1^ *p*-Value was obtained and calculated using ANOVA or a Chi-squared test.

**Table 6:** (a) Infant anthropometry and impact of wasting; (b) infant anthropometry and impact of wasting.

(a)						
	Real Child Height					
	Total (n = 437) Frequency (%) Mean ± SD	Stunted (n = 251) Frequency (%) Mean ± SD	Not Stunted (n = 186) Frequency (%) Mean ± SD	*p*-Value ^1^	PR	95% CI
Weight (kg)	7.3 ± 1.7	7.2 ± 1.6	7.5 ± 1.9	0.033		
Height (cm)	66.2 ± 8.5	65.2 ± 7.9	67.6 ± 9.1	0.003		
Difference	−5.2 ± 5.5	−7.7 ± 3.2	−1.6 ± 3.3	<0.001		
Weight-for-height (WFH)	−0.01 ± 1.85	−0.09 ± 2.01	−0.16 ± 1.60	0.157		
<−3 SD Severe wasting	13 (3.0%)	8 (3.2%)	5 (2.7%)	0.135	1.28	0.41–4.02
≥−3 to - ≤−2 SD Moderate wasting	41 (9.4%)	24 (9.5%)	17 (9.2%)		1.13	0.58–2.20
>−2 to ≤+1 SD Normal	283 (64.8%)	157 (62.3%)	126 (68.1%)		1 Reference	-
>+1 to ≤+2 SD Overweight risk	42 (9.6%)	22 (8.7%)	20 (10.8%)		0.88	0.46–1.69
≥+2–≤+3 SD Overweight	26 (5.9%)	15 (6.0%)	11 (5.9%)		1.09	0.48–2.46
>+3 SD Obesity	32 (7.3%)	26 (10.3%)	6 (3.2%)		2.86	1.20–6.84
(**b**)						
	**Adjusted to Who Height-for-Age**					
	**Total** **(n = 437)** **Frequency (%)** **Mean ± SD**	**Stunted** **(n = 251)** **Frequency (%)** **Mean ± SD**	**Not Stunted** **(n = 186)** **Frequency (%)** **Mean ± SD**	***p*-Value ^1^**	**PR**	**95% CI**
Weight (kg)	-	-	-			
Height (cm)	71.4 ± 9.6	73.1 ± 8.9	69.1 ± 9.9	<0.001		
Difference	-	-	-			
Weight-for-height (WFH)	−1.7 ± 2.3	−2.5 ± 2.2	−0.5 ± 1.8	<0.001		
<−3 SD Severe wasting	119 (27.2%)	107 (42.6%)	12 (6.5%)	<0.001	13.11	6.80–25.30
≥−3 to - ≤−2 SD Moderate wasting	66 (15.1%)	44 (17.5%)	22 (11.8%)		2.94	1.64–5.26
>−2 to ≤+1 SD Normal	210 (48.1%)	85 (33.9%)	125 (67.2%)		1 Reference	-
>+1 to ≤+2 SD Overweight risk	23 (5.3%)	7 (2.8%)	16 (8.6%)		0.64	0.25–1.63
≥+2–≤+3 SD Overweight	8 (1.8%)	4 (1.6%)	4 (2.2%)		1.47	0.36–6.04
>+3 SD Obesity	11 (2.5%)	4 (1.6%)	7 (3.8%)		0.84	0.24–2.96

SD: standard deviation; PR: prevalence ratio; ^1^ *p*-Value was obtained and calculated using ANOVA or a Chi-squared test.

**Table 7:** Theoretical impact of wasting.

Real Height	Adjusted to WHO Height-for-Age						
	<−3 SD Severe Wasting	≥−3 to ≤−2 SD Moderate Wasting	>−2 to ≤+1 SD Normal	>+1 to ≤+2 SD Overweight Risk	≥+2–≤+3 SD Overweight	>+3 SD Obesity	*p*-Value ^1^
	Frequency (%) Mean ± SD	Frequency (%) Mean ± SD	Frequency (%) Mean ± SD	Frequency (%) Mean ± SD	Frequency (%) Mean ± SD	Frequency (%) Mean ± SD	
Theoretical WFH classification	119 (27.2%)	66 (15.1%)	210 (48.1%)	23 (5.3%)	8 (1.8%)	11 (2.5%)	
Real WFH classification	13 (3.0%)	41 (9.4%)	283 (64.8%)	42 (9.6%)	26 (5.9%)	32 (7.3%)	
<−3 SD Severe wasting	10 (2.3%)	2 (0.5%)	1 (0.2%)	0 (0%)	0 (0%)	0 (0%)	<0.001
≥−3 to ≤−2 SD Moderate wasting	25 (5.7%)	7 (1.6%)	9 (2.1%)	0 (0%)	0 (0%)	0 (0%)	
>−2 to ≤+1 SD Normal	78 (17.8%)	55 (12.6%)	141 (32.3%)	5 (1.1%)	2 (0.5%)	2 (0.5%)	
>+1 to ≤+2 SD Overweight risk	3 (0.7%)	1 (0.2%)	29 (6.6%)	7 (1.6%)	1 (0.2%)	1 (0.2%)	
≥+2–≤+3 SD Overweight	3 (0.7%)	1 (0.2%)	17 (3.9%)	1 (0.2%)	1 (0.2%)	3 (0.7%)	
>+3 SD Obesity	0 (0%)	0 (0%)	13 (3.0%)	10 (2.3%)	4 (0.9%)	5 (1.1%)	

SD: standard deviation; ^1^ *p*-Value was obtained and calculated using ANOVA or a Chi-squared test.

## Data Availability

The datasets presented in this article are not readily available due to ethical personal data sharing restrictions. Requests to access the datasets should be directed to the corresponding author.
